# TGF-β-regulated different iron metabolism processes in the development and cisplatin resistance of ovarian cancer

**DOI:** 10.32604/or.2023.031404

**Published:** 2023-12-28

**Authors:** JIANFA WU, QIANYI LIAO, LI ZHANG, SUQIN WU, ZHOU LIU

**Affiliations:** 1 Department of Gynecology, Shanghai University of Medicine & Health Sciences Affiliated Zhoupu Hospital, Shanghai, China; 2 Department of Gynecology, Shanghai University of Medicine & Health Sciences, Shanghai, China; 3 Department of Gynecology, Gongshan People’s Hospital, Nujiang Lisu Autonomous Prefecture, China

**Keywords:** Chemoresistance, Cisplatin, Iron, Ovarian neoplasms, TGF-β

## Abstract

The impact of different iron metabolism processes (DIMP) on ovarian cancer remains unclear. In this study, we employed various gene chips and databases to investigate the role of DIMP in the initiation and development of ovarian cancer. cBioPortal was used to determine mutations in DIMP-associated genes in ovarian cancer. Kaplan-Meier plotter was used to examine the influence of DIMP on the prognosis of ovarian cancer. By analyzing 1669 serous ovarian cancer cases, we identified a range of mutations in iron metabolism genes, notably in those coding for the transferrin receptor (19%), melanotransferrin (19%), and ceruloplasmin (10%) in the iron import process, and glucose-6-phosphate isomerase (9%), hepcidin antimicrobial peptide (9%), metal regulatory transcription factor 1 (8%), and bone morphogenetic protein 6 (8%) in the iron regulation process. Compared to the unaltered group, the group with gene alterations exhibited a higher tumor mutation burden count (43 *vs*. 54) and more advanced histologic grade (78.19% *vs*. 87.90%). Compared to the normal ovarian counterparts, a reduction in expression was observed in 9 out of the 14 genes involved in iron utilization and 4 out of the 5 genes involved in iron export in ovarian cancer; in contrast, an increase in expression was observed in 2 out of the 3 genes involved in iron storage in ovarian cancer. Furthermore, in cisplatin-resistant cells compared to cisplatin-sensitive ones, the expression of all genes in iron storage and 13 out of 14 genes in iron import was decreased, while that of 8 out of the 10 genes in iron utilization was increased. In addition, survival curve analysis indicated that a higher expression in the majority of genes in the iron import process (12/21), or a reduced expression in most genes in the iron export process (4/5) correlated with poor progression-free survival. Additionally, TGF-β could regulate the expression of most iron metabolism-associated genes; particularly, expression of genes involved in the iron storage process (2/2) was inhibited after TGF-β1 or TGF-β2 treatment. In conclusion, DIMP plays multifaceted roles in the initiation, chemo-resistance, and prognosis of ovarian cancer. Therapeutically targeting DIMP may pave the way for more tailored treatment approaches for ovarian cancer.

## Introduction

With the advancement of high-throughput sequencing and molecular targeted therapies, aberrant gene expression has been linked to the emergence of ovarian cancer and cisplatin resistance [1]. Yet, despite being one of the three primary malignancies of the female genital system, the underlying cause and mechanisms of drug resistance in ovarian cancer remain elusive; in addition, the 5-year survival rate for ovarian cancer remains relatively low (~45%) [2]. Extensive studies underscore that the development of ovarian cancer is a complex process involving numerous genes.

Iron, as an important element within the human body, plays vital roles beyond its well-known contribution to hemoglobin. It forms an integral part of various enzymes and facilitates multiple biological processes, such as energy metabolism, hemoglobin production, DNA synthesis, and immune regulation [3]. Previous studies suggest that deviations in iron content can lead to disorders such as anemia, atherosclerosis, cognitive impairment, pre-eclampsia, and polycystic ovary syndrome [4–6]. Furthermore, emerging research underscores the ties between dysregulated iron metabolism and cancer [7], with a notable emphasis on its linkage to ovarian cancer. Excessive iron is believed to influence tumor onset, tumor metastasis, and drug resistance [8,9]. *In vitro* studies have identified an iron chelator that can sensitize ovarian cancer to cisplatin [9]. The augmented iron levels in the pelvic cavity—attributed to menstrual blood, ovulation, and follicular fluid—are hypothesized to be crucial in the initiation and development of endometrioid and clear-cell ovarian cancers [10]. Yet, the specific mechanism underlying iron overload in ovarian cancer remains enigmatic. One study suggested that iron overload contributes to cancer development by activating oxidative stress and causing gene mutations [11]. An increase in reactive oxygen species can lead to DNA damage, subsequently inhibiting the P53/phosphatase and tensin homolog signaling pathway while activating the mitogen-activated kinase-like protein signaling pathway, boosting tumor genesis [12]. However, many studies have honed on the impact of individual iron metabolism genes on ovarian cancer, often ignoring the overall interplay between different iron metabolism processes (DIMP) and ovarian disease. This narrow lens has somewhat hindered our comprehensive understanding of ovarian cancer.

Based on previous studies, iron metabolism encompasses five processes: iron import, iron regulation, iron export, iron storage, and iron utilization [13]. Numerous iron regulatory genes participate in these different DIMP. Although these processes function autonomously, they collectively maintain iron homeostasis. However, the implications of DIMP in the development, drug resistance, and prognosis of ovarian cancer remain unclear. Further understanding on DIMP’s influence on ovarian cancer is paramount for tailoring precise treatments and minimizing adverse outcomes. This study sets out to elucidate the multifaceted roles of DIMP in ovarian cancer.

## Iron Import

Previous studies have demonstrated that a myriad of genes participate in iron import. The duodenum primarily absorbs non-heme iron, with minimal absorption from the stomach and intestine. Within the duodenum, Fe^3+^ is reduced to Fe^2+^ by Duodenal Cytochrome B [14], a protein encoded by CYBRD1 that is predominantly expressed in the duodenal brush border membrane. The Fe^2+^ is then taken up by the divalent metal ion transporter 1 (SLC11A2) [15]. Subsequently, solute carrier family 40 member 1 (SLC40A1) facilitates the movement of Fe^2+^ across membranes into the bloodstream. Ceruloplasmin (CP) and hephaestin (HEPH) then oxidize Fe^2+^ back to Fe^3+^. Notably, CP levels in ascites were observed to be higher in chemoresistant serous epithelial ovarian cancer (EOC) patients compared to chemosensitive serous EOC patients [16]. Post-carboplatin treatment, patients with lower CP levels tend to have enhanced therapeutic responses [17]. HEPH, a member of the multicopper oxidase protein family, aids in transporting dietary iron from the epithelial cells of the intestinal lumen into the circulatory system [18]. Once in circulation, Fe^3+^ binds to transferrin (TF) for transportation. While a significant portion of Fe^3+^ is transported to the bone marrow, some get engulfed and stored in macrophages. The remaining Fe^3+^ binds to cell surface receptors, transferrin receptor 1 (TFR1) or transferrin receptor 2 (TFR2), and is reduced back to Fe^2+^ by STEAP3 metalloreductase (STEAP3) and STEAP2 metalloreductase (STEAP2) [19]. Additionally, lipocalin 2 (LCN2) facilitates the transportation of catechol-bound iron from the extracellular environment to the cytoplasm, affecting the expression of ferritin and TFR1 [20]. Acting as the receptor of LCN2, solute carrier family 22 member 17 (SLC22A17) is believed to contribute to siderophore transport to maintain iron homeostasis [21]. The LCN2/SLC22A17 complex promotes tumor proliferation, which can be curbed by iron chelators [22].

The transport of heme iron involves several carrier families: solute carrier family 46 member 1 (SLC46A1), solute carrier family 48 member 1 (SLC48A1), solute carrier family 25 member 28 (SLC25A28), and solute carrier family 25 member 37 (SLC25A37). Specifically, SLC46A1 functions as a heme importer within duodenal enterocytes and is influenced by dietary iron levels [23]. Additionally, SLC48A1 serves as a heme transporter, responsible for heme binding activity and heme transmembrane transporter activity [24]. Both SLC25A28 and SLC25A37, situated in the mitochondrial inner membrane, play vital roles as iron importers, contributing to the synthesis of mitochondrial heme and iron-sulfur clusters [25].

Numerous other iron-associated genes also play a role in the iron import process. For instance, Lactotransferrin (LTF), a member of the transferrin family, not only regulates iron transport but also has roles in combating inflammation, microbial infection, cancer development, and metastasis [26]. The lactoferrin receptor specifically promotes iron absorption [27]. The solute carrier family 39 member 14 (SLC39A14) plays a crucial role in transporting non-transferrin-bound iron, and it also mediates the cellular uptake of other metals like manganese, zinc, iron, and cadmium [28]. Melanotransferrin (MELTF), a cell-surface glycoprotein, has similarities in sequence and iron-binding properties with the transferrin superfamily, though its exact role remains elusive. Additionally, poly-(rC)-binding protein 1 (PCBP1) and poly-(rC)-binding protein 2 (PCBP2) are crucial for iron transport. They bind to the labile iron pool (LIP) to deliver the metal to the iron storage protein ferritin [29].

## Iron Export

Previously, SLC40A1 was believed to be the sole iron exporter [30]. A reduced expression of SLC40A1 results in iron overload in ovarian cancer cells, which has been linked to cisplatin resistance [8]. MON1 homolog A, secretory trafficking associated (MON1A) transports intracellular SLC40A1 to the plasma membrane, where SLC40A1 can act as an iron exporter [31]. Once exported, the iron is oxidized by either CP or HEPH before being loaded onto TF.

The feline leukemia virus C receptor (FLVCR) has been identified as a heme exporter. FLVCR encodes a heme transporter that is important in erythropoiesis [32]. ATP-binding cassette subfamily G member 2 (ABCG2) is another heme exporter with a critical role in multi-drug resistance. Notably, ABCG2 interacts with heme and porphyrin, facilitating their exocytosis [33].

## Iron Storage

Ferritin serves as the primary storage for excess ferrous iron. Comprising 24 subunits, ferritin consists of the ferritin heavy chain (FTH) and ferritin light chain (FTL) [34]. A single ferritin molecule can bind up to 4,500 iron atoms. By binding to free iron, ferritin prevents cells from iron-dependent peroxidation damage. Ferritin within the mitochondria enhances the ferric iron binding activity, which is crucial for maintaining iron homeostasis [35]. Furthermore, the mitochondria also act as a regulator controlling the amount of free ferrous iron present in the cell.

## Iron Utilization

Iron is primarily utilized in the mitochondria for heme synthesis, a process facilitated by numerous proteins. In the heme biosynthetic pathway, 5′-aminolevulinate synthase 2 (ALAS2) catalyzes the first step [36]. The enzyme 5′-aminolevulinate synthase 1 (ALAS1), encoded by mitochondrial DNA, catalyzes the rate-limiting step in heme (iron-protoporphyrin) biosynthesis, with its activity regulated by heme levels: a low heme level up-regulate and a higher heme level downregulates it [37]. The next step involves the enzyme aminolevulinate dehydratase (ALAD), comprising eight identical subunits [38]. Following that, hydroxymethylbilane synthase (HMBS), a member of the hydroxymethylbilane synthase superfamily, catalyzes the third step, while uroporphyrinogen III synthase (UROS) catalyzes the fourth step [39]. Then, uroporphyrinogen decarboxylase (UROD) catalyzes the conversion of uroporphyrinogen to coproporphyrinogen by excising four carboxymethyl side chains [40]. Protoporphyrinogen oxidase (PPOX) then catalyzes the transformation of protoporphyrinogen IX into protoporphyrin IX [41]. Ferrochelatase (FECH) subsequently mediates the integration of ferrous iron into protoporphyrin IX [42]. Meanwhile, coproporphyrinogen oxidase (CPOX) plays a significant role in the transformation of coproporphyrinogen III to protoporphyrinogen IX [43]. Lastly, the ATP-binding cassette subfamily B member 6 (ABCB6) aids in the translocation of coproporphyrinogen III from the cytoplasm to mitochondria [44].

The second way of iron being used is the formation of iron-sulfur clusters, with numerous proteins involved. Iron-sulfur cluster assembly enzyme (ISCU), as a component of the iron-sulfur (Fe-S) cluster scaffold, plays a vital role in regulating metabolism, maintaining iron homeostasis, and responding to oxidative stress [45]. Iron-sulfur cluster assembly 1 (ISCA1), a protein localized in the mitochondria, contributes to the biogenesis and assembly of these clusters [45]. Cytosolic iron-sulfur protein assembly 1 (CIAO1) also aids in both iron-cluster assembly and protein maturation through iron-sulfur cluster transfer [45]. In addition, ABCB7 plays an essential role in the formation of iron-sulfur clusters, transporting them from mitochondria to the cytoplasm [46].

Many other genes also contribute to the formation of heme and iron-sulfur clusters. Frataxin (*FXN*) encodes a mitochondrial protein that belongs to the FXN family. This protein has a central role in modulating mitochondrial iron transport and respiration, both of which are essential for iron-sulfur cluster formation and heme biosynthesis [47]. Additionally, iron-sulfur cluster assembly proteins receive iron bound to FXN, a crucial step in forming iron-sulfur clusters [48].

## Iron Regulation

Iron metabolism in cells is intricately controlled by various factors, with the iron-regulatory protein playing a pivotal role in maintaining iron balance. The iron-regulatory protein consists of two family members: iron-regulatory protein 1 (ACO1) and iron-responsive element-binding protein 2 (IREB2). Under low iron conditions, ACO1 and IREB2 are activated and bind to IRE in the 5′ UTR of FTH, FTL, SLC40A1, and HIF 2α to increase iron levels. Moreover, they also bind to the IRE in the 3′ UTR of SLC11A2 and TFR1 to maintain iron homeostasis in cells [49]. Notably, binding to the 5′ UTR suppresses translation, while binding to 3′ UTR prevents mRNA degradation [50]. In conditions of ample iron, IREB2 is degraded and ACO1’s IRE binding site is occupied by iron-sulfur clusters, thus deactivating its function.

Hepcidin antimicrobial peptide (HAMP) is another crucial iron regulator for iron homeostasis. Hepatic iron storage, inflammation, erythropoietic activity, and expression of HAMP can also be affected by iron overload [51]. HAMP inhibits the release of iron from duodenal enterocytes, macrophages, and hepatocytes. Furthermore, HAMP binds to SLC40A1 to facilitate its degradation in lysosomes [52]. In addition, the bone morphogenetic protein (BMP) family and other members of the TGF family transcriptionally control HAMP expression [53]. Notably, bone morphogenetic protein 6 (BMP6), a secreted ligand of the TGF-β superfamily proteins, binds to the BMP receptor and hemojuvelin (HJV). This binding enhances the phosphorylation of SMAD family member 4 (SMAD4) and the expression of HAMP to maintain iron homeostasis [54].

Hypoxia-inducible factor 1 (HIF 1) and hypoxia-inducible factor 2 (HIF 2) proteins play essential roles in iron homeostasis regulation. HIF 1α is essential for the expression of iron regulatory proteins, especially for HAMP, heme oxygenase 1 (HMOX-1), TFR, and CP [55]. This underscores the importance of iron in sustaining the homeostasis and viability of HIF. In contrast, HIF 2α, by enhancing FXN expression, facilitates the formation of iron-sulfur clusters and heme [56]. The levels of both HIF 1 and HIF 2 are modulated by the prolyl hydroxylase domain (PHD). As a 2-oxoglutarate-dependent dioxygenase, PHD’s activity hinges on the availability of iron and oxygen. It enhances HIF hydroxylation, triggering HIF’s degradation via ubiquitination. Notably, factor inhibiting hypoxia-inducible factor 1 (FIH1), a member of the PHD family, regulates the HIF activity through asparaginyl hydroxylation [57].

Numerous genes have been identified as key players in iron regulation. Glucose-6-phosphate isomerase (GPI) is identified as a moonlighting protein, given its multifaceted functions. Specifically, GPI-linked CP protects ferroportin from internalization and degradation [58]. Moreover, GPI-linked HJV activates the BMP signaling cascade to modulate hepcidin transcription [59]. Homeostatic iron regulator (HFE) controls iron absorption by regulating transferrin receptor and transferrin interaction [45]. HFE interacts with TFR2 to promote hepcidin transcription under high concentrations of holo-Tf [60]. Metal regulatory transcription factor 1 (MTF1), as a transcription factor, induces the expression of metallothioneins and other genes involved in metal homeostasis. It also up-regulates ferritin/FPN1 and inhibits ferroptosis [61]. Recent studies demonstrated that depletion of sirtuin 2 leads to diminished cellular iron levels [62]. Both HMOX1 and heme oxygenase 2 (HMOX2) are vital in heme catabolism, cleaving heme to produce biliverdin. This biliverdin is subsequently converted into bilirubin through biliverdin reductase and carbon monoxide [63].

## Materials and Methods

### Expression analysis

The expression of different genes was compared using 376 ovarian cancers from TCGA dataset (https://portal.gdc.cancer.gov/) and 180 ovary tissues from Genotype-Tissue Expression (GTEx) dataset (https://commonfund.nih.gov/GTex) [64].

### Gene microarray analysis

Two gene microarrays (GDS3754, GDS5351) were employed in this study [65], using the platforms GPL570 and GPL8341. Expression data and promoter CpG island methylation data from A2780 (cisplatin-sensitive) and A2780CP (cisplatin-resistant) cell lines were compared. GDS5351 was obtained from the platform GPL570. After treatment with 5 ng/ml TGF-β1 or TGF-β2 for 48 h, total RNA was extracted from human telomerase-immortalized ovarian fibroblast line NOF151. After cDNA synthesis, *in vitro* transcription, and biotin labeling, the gene expression data were obtained; the data between TGF-β1 group or TGF β2 group and the control group were compared [66].

### Survival analysis using Kaplan–Meier plotter

Gene expression data and survival information of the patients in the Kaplan Meier plotter were obtained from GEO, EGA, and TCGA dataset (54,000 genes, and 21 cancer types). Most tumor sample data were for ovarian cancer, lung cancer, gastric cancer, and breast cancer. A PostgreSQL server was employed to analyze all data, which determined the prognostic value of various genes in tumor patients based on gene expression level. The hazard ratio was calculated using log-rank *p* value and 95% confidence intervals [67]. The research project protocol had been approved by Zhoupu Hospital Ethics Committee and it conformed to the provisions of the Declaration of Helsinki in 1995. Informed consent were obtained from all patients.

### Mutation analysis of iron metabolism-associated genes in ovarian cancer

Iron metabolism-associated gene data were obtained from CBioPortal (http://www.cbioportal.org/). Genomic alterations, including amplifications, structural variants, splice mutations, missense mutations, truncating mutations, and deep deletions in 1669 serous ovarian cancer cases were explored. Furthermore, all patients were categorized into two groups based on gene mutation: the altered group (n = 587) and the unaltered group (n = 1082). Variables, including age, fraction genome altered, mutation count, TMB, histologic grade, MSIsensor score, primary therapy outcome (surgery or chemotherapy), lymphovascular invasion, tumor stage, neoadjuvant therapy (carboplatin and paclitaxel), and race, were compared between the altered group and unaltered group.

### Analysis of the relationship between iron metabolism-associated genes and stage of ovarian cancer

The GEPIA database (http://gepia.cancer-pku.cn/) was used to explore the relationship between different iron metabolism-associated genes and stages of ovarian cancer [68]. All tumor gene expression data used in the analyses were obtained from the TCGA databases.

### Establishment of a prognosis model of ovarian cancer with different iron metabolism-associated genes

RNA-sequencing expression profiles and corresponding clinical information of 376 patients were obtained from the TCGA dataset (https://portal.gdc.cancer.gov/). Differences in survival between various groups were compared using the Log-rank test. Furthermore, the timeROC (v 0.4) analysis was employed to compare the predictive accuracy of various molecular models and risk scores [69].

### Statistics analysis

Measurement data were analyzed using *t*-test, calibration *t*-test, variance analysis, or Wilcoxon test via SPSS 22. Normally distributed data were presented as x ± s. The counting data were analyzed using the chi-square test, chi-square test of continuous correction, or Fisher’s exact probability method. GraphPad Prism 6.0 was used to generate the statistical charts.

## Result

### Mutation of iron metabolism-associated genes in ovarian cancer

**Figure 1:**
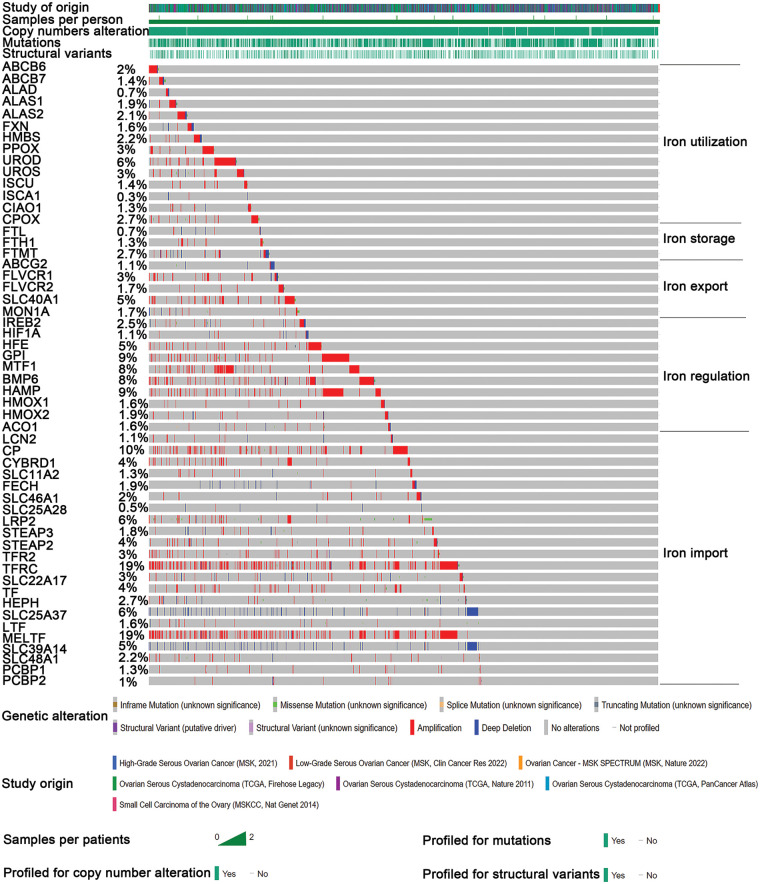
Mutation analysis of iron metabolism-associated genes in ovarian cancer. Seven different studies were employed to analyze mutations in various iron metabolism-associated genes in ovarian cancer. Iron metabolism processes were divided into five processes. Mutations of various iron metabolism genes in five different iron metabolism processes (DIMP) were examined in 1669 cases of ovarian cancer using the CBioPortal database. Different colors represent different types of genomic alterations.

Through detailed mutation analysis, a spectrum of genomic alterations, including amplifications, structural variants, splice mutations, missense mutations, truncating mutations, and deep deletions, were detected in DIMP-related genes, particularly genes encoding transferrin receptor (*TFRC*) (19%), *MELTF* (19%), *CP* (10%) in the iron import process, and *GPI* (9%), *HAMP* (9%), *MTF1* (8%), and *BMP6* (8%) in the iron regulation process (Fig. 1). When compared to the unaltered group, the altered group demonstrated higher values in metrics like genome mutation count, genome tumor mutation burden count (TMB), MSIsensor score, and fraction genome alteration (Table 1). Post primary treatment, the disease progression rate was marginally higher in the altered group than in the unaltered group (9.73% & 8.88%, Table 1). Moreover, ovarian cancer cases in the altered group were more likely to be presented as poorly differentiated (G3) or undifferentiated (G4) when compared to the unaltered group (Table 1). The incidence of lymphovascular invasion was also higher in the altered group than the unaltered group (67.1% & 50%, Table 1). These findings underscore the prevalence of mutations in genes associated with iron metabolism, particularly in iron import and regulation processes, in ovarian cancer. The mutations in these iron metabolism-associated genes lead to genome instability, as indicated by increased TMB and microsatellite stability (MSI); these factors possibly contribute to the initiation of ovarian cancer. Importantly, these mutations correlate with lymphovascular invasion, unfavorable pathological grades, and poor treatment outcomes. This suggests that dysregulated iron metabolism could be a significant factor contributing to the poor prognosis of ovarian cancer.

**Table 1:** Relationship between iron metabolism-associated genes and clinical characteristics

Clinical attribute	Attribute type	Statistical test	Altered group (587)	Unaltered group (1082)	*p* value	q-value
**Age**	Patient	Wilcoxon test	59(37–89)	59(20.5–87)	0.18	0.59
**Fraction genome altered**	Sample	Wilcoxon test	0.59(0.09–1)	0.49(0.01–1)	1.51e-10	5.98e-9
**Mutation count**	Sample	Wilcoxon test	54(2–151)	43(1–115.5)	1.89e-9	4.99e-8
**TMB**	Sample	Wilcoxon test	1.67(0–4.9)	1.27(0–3.98)	2.00e-8	2.64e-7
**Histologic grade**	Patient/sample	Chi-squared test			2.54e-5	2.51e-4
G1			0	3.64%		
G2			10.67%	14.55%		
G3			87.9%	77.58%		
G4			0	0.61%		
GB			0.25%	0.61%		
GX			1.23%	3.03%		
**MSIsensor score**	Sample	Wilcoxon test	0.89(0–3)	0.67(0–2.57)	2.88e-4	2.53e-3
**Primary therapy outcome**	Patient	Chi-squared test			0.02	0.13
Complete response			74.34%	63.91%		
Partial response			12.39%	17.16%		
Progressive disease			9.73%	8.88%		
Stable disease			3.54%	10.06%		
**Lymphovascular invasion**	Patient	Chi-squared test			0.03	0.14
Yes			67.1%	50%		
No			32.9%	50%		
**Tumor stage**	Sample	Chi-squared test			0.06	0.26
II			6.31%	3.02%		
III			74.73%	84.43%		
IV			18.95%	12.56%		
**Neoadjuvant therapy**	Patient	Chi-squared test			0.18	0.59
Yes			0	0.61%		
No			100%	99.39%		
**Race**	Patient	Chi-squared test			0.13	0.52
American Indian or Alaska			0.72%	0.33%		
Asian			3.62%	3.61%		
Black or African-American			7.38%	4.26%		
Hawaiian or other pacific islander			0.14%	0		
White			88.13%	91.8%		

### Relationship between different iron metabolism processes and initiation of ovarian cancer

**Figure 2:**
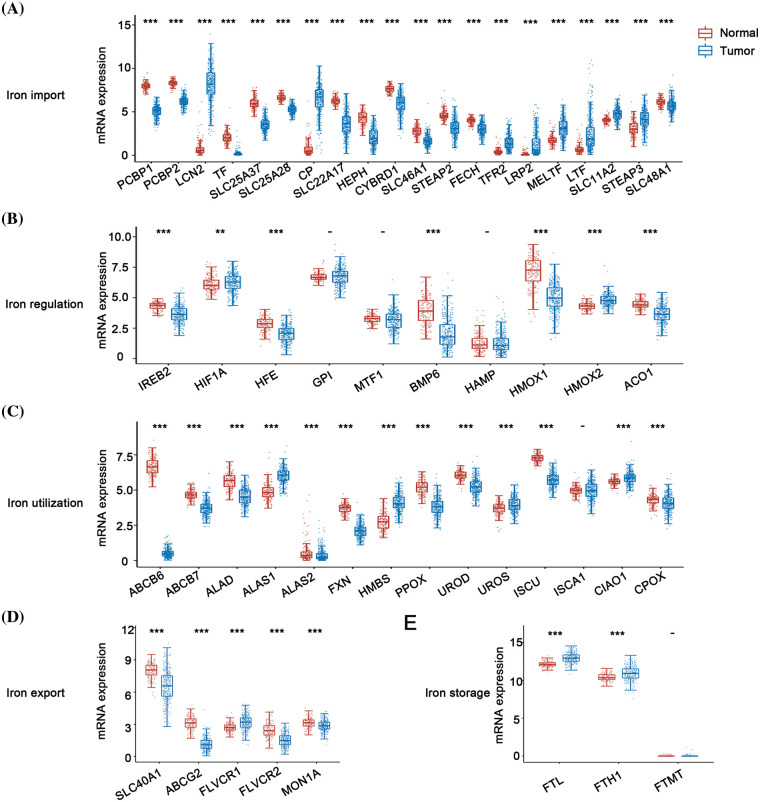
Expression analysis of different iron metabolism-associated genes in ovarian cancer. 376 ovarian cancer and 180 ovary tissue from The Cancer Genome Atlas (TCGA) were used to investigate the expression of various iron metabolism-associated genes. The horizontal axis denotes different genes, the vertical axis denotes the expression distribution of related genes, and different colors represent various groups. (A) The mRNA expression of various genes in the iron import process between normal ovarian tissue and ovarian cancer. (B) The mRNA expression of various genes in the iron regulation process between normal ovarian tissue and ovarian cancer. (C) The mRNA expression of various genes in the iron utilization process between normal ovarian tissue and ovarian cancer. (D) The mRNA expression of various genes in the iron export process between normal ovarian tissue and ovarian cancer. (E) The mRNA expression of various genes in the iron storage process between normal ovarian tissue and ovarian cancer. **p < 0.01, ***p < 0.01.

This study discussed the relationship between DIMP-related genes and the initiation of ovarian cancer. Intriguingly, ovarian cancer tissues exhibited decreased expression of 12 genes pivotal in iron import compared to normal tissues, including *PCPB1*, *PCPB2*, *TF*, *SLC25A37*, *SLC25A28*, *SLC22A17*, *HEPH*, *CYBRD1*, *SLC46A1*, *STEAP2*, *FECH*, and *SLC48A1*. Conversely, 8 genes showed increased expression in ovarian cancer tissues, including *LCN2*, *CP*, *TFR2*, *LRP2*, *MELTF*, *LTF*, *SLC11A2*, and *STEAP3* (Fig. 2A). However, the causal relationship between iron import and initiation of ovarian cancer remains enigmatic. Exploring the iron regulation in ovarian cancer, most genes integral to this process, like *IREB2*, *HFE*, *BMP6*, *HMOX1*, and *ACO1*, showed decreased expression compared to their normal tissue counterparts (Fig. 2B). In contrast, the expression of *HIF1A* and *HMOX2* involved in iron regulation was found to be higher in ovarian cancer tissues (Fig. 2B). Based on these data, one can postulate that suppressed iron regulation may play a role in triggering ovarian cancer. The role of iron utilization became evident when most genes critical to this process, such as *ABCB6*, *ABCB7*, *ALAD*, *ALAS2*, *FXN*, *PPOX*, *UROD*, *ISCU*, *CPOX*, showed a decreased expression in cancerous tissues (Fig. 2C). In contrast, genes like *ALAS1*, *HMBS*, *UROS*, *CIAO1* showed an opposite trend (Fig. 2C). These findings suggest that reduced iron utilization may contribute to the initiation of ovarian cancer. Similarly, most genes involved in iron export, such as *SLC40A1*, *FLVCR1*, *ABCG2*, and *MON1A*, displayed reduced expression in ovarian cancer tissues; in contrast, only one gene, *FLVCR2*, showed the opposite trend (Fig. 2D). These findings indicate a potential link between iron overload and the initiation of ovarian cancer. Lastly, most genes (*FTL* and *FTH1*) involved in the iron storage process displayed increased expression in ovarian cancer tissues (Fig. 2E), suggesting an association between this process and the initiation of ovarian cancer.

### Relationship between different iron metabolism processes and stages of ovarian cancer

**Figure 3:**
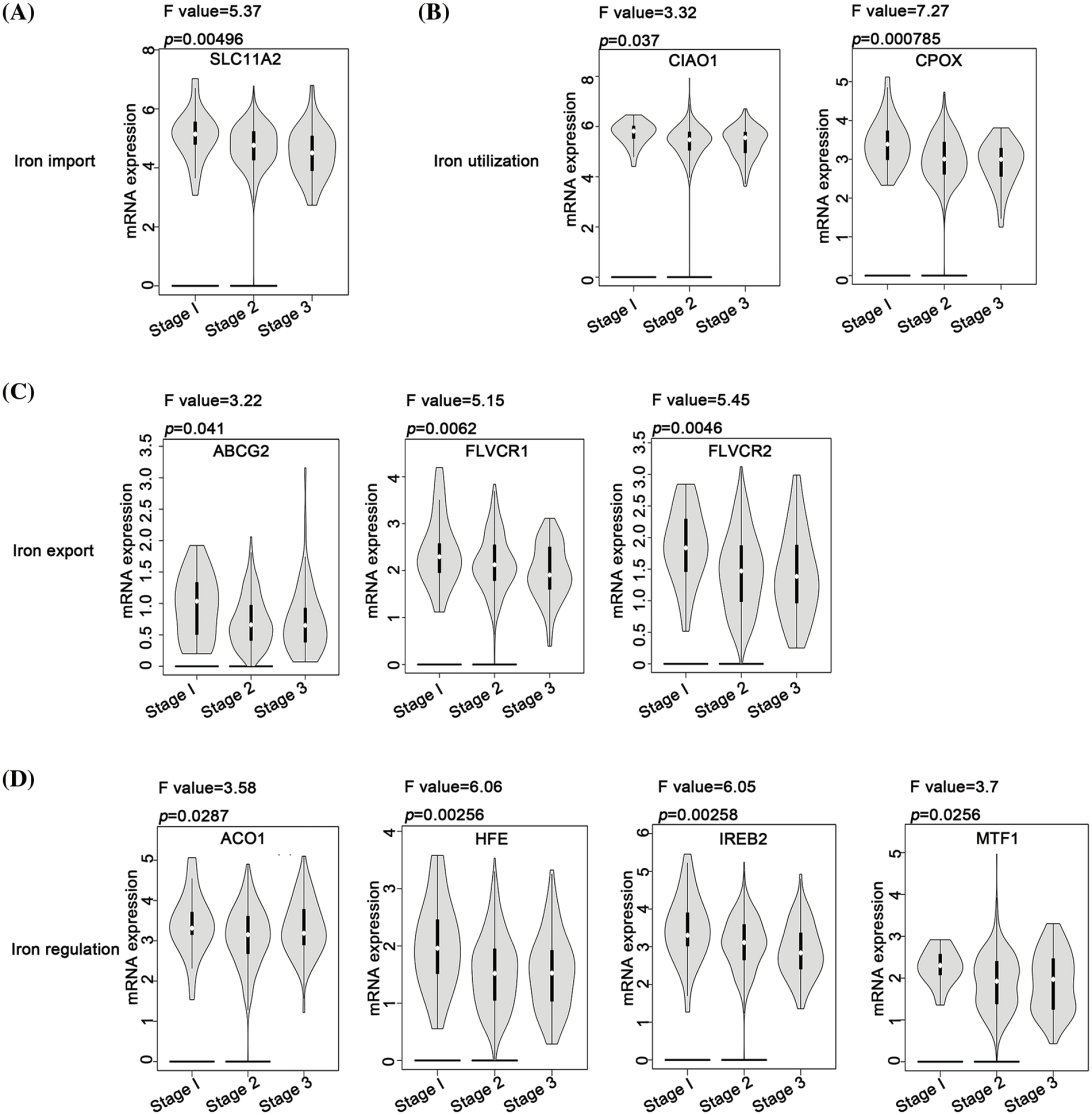
Analysis of the relationship between different iron metabolism-associated genes and stage of ovarian cancer. With the GEPIA database, mRNA expression of DIMP-associated genes was compared among patients with stage 1, stage 2, and stage 3, including (A) iron import, (B) iron utilization, (C) iron export, and (D) iron regulation. The horizontal axis represents different genes, and the vertical axis represents the expression distribution of related genes.

In the context of iron import, except for *SLC11A2*, most genes did not exhibit a significant correlation with the varying stages of ovarian cancer (Fig. 3A). For iron utilization, a lower expression of *CIAO1* and *CPOX* was associated with a more advanced stage of ovarian cancer (Fig. 3B). Similarly, for iron export, lower expression levels of *ABCG2*, *FLVCR1*, and *FLVCR2* were associated with an advanced stage of ovarian cancer (Fig. 3C). Furthermore, lower expression levels of *ACO1*, *HFE*, *IREB2*, and *MTF1* in iron regulation were associated with an advanced stage of ovarian cancer (Fig. 3D). Contrastingly, the iron storage process did not present any genes that could be linked to the pathological stage of ovarian cancer. These observations suggest that inhibition of the iron export and iron regulation processes might be associated with ovarian cancer’s progression. In contrast, iron import, iron utilization, and iron storage processes might have limited influence on the disease’s progression.

### Relationship between different iron metabolism processes and cisplatin resistance in ovarian cancer

**Figure 4:**
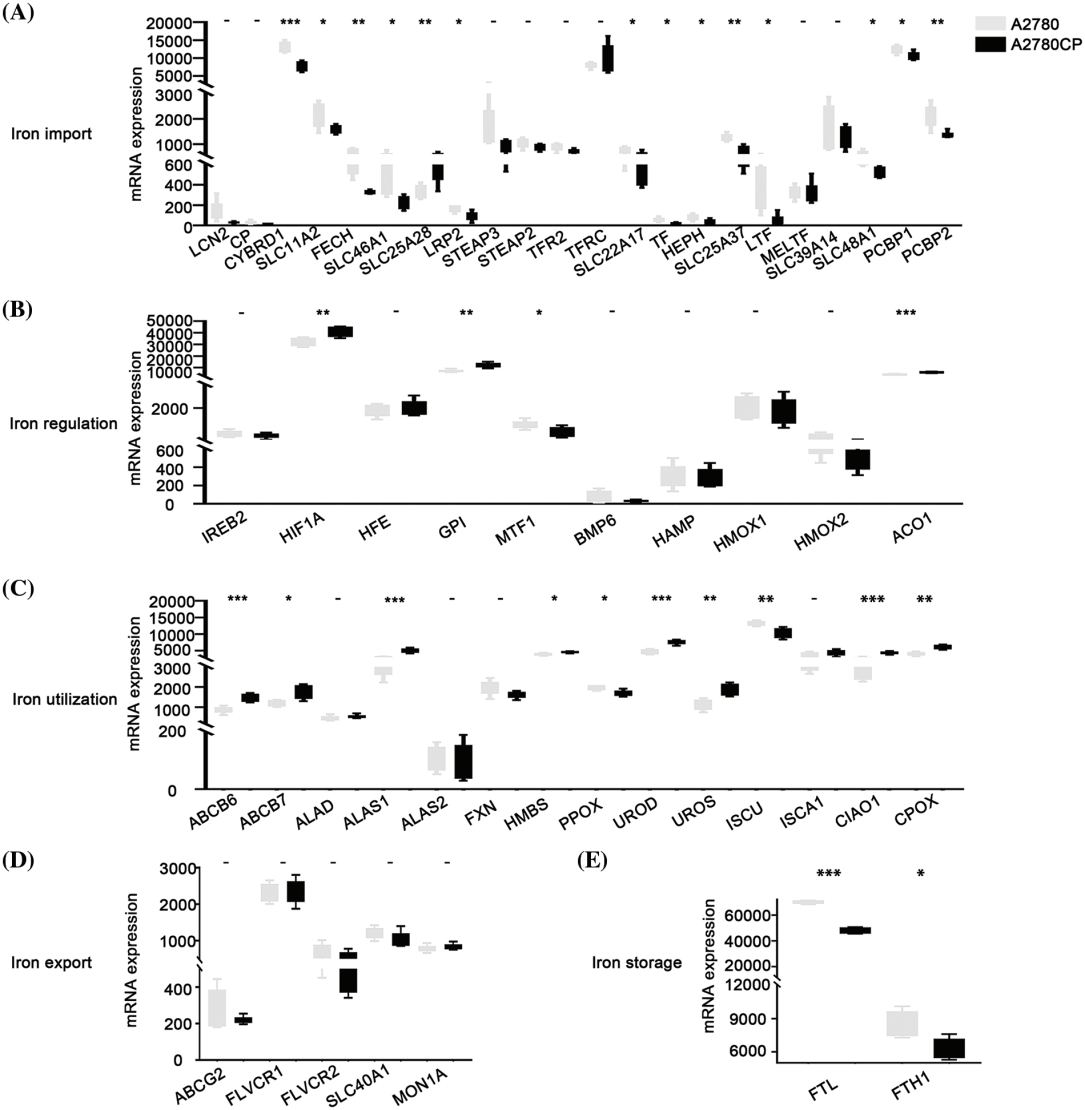
Analysis of the relationship between different iron metabolism-associated genes and cisplatin resistance in ovarian cancer. mRNA expression of DIMP-associated genes was compared between A2780 (cisplatin sensitive) and A2780CP (cisplatin resistance), including (A) iron import, (B) iron regulation, (C) iron utilization, (D) iron export, and (E) iron storage. The horizontal axis represents different genes, the vertical axis represents the expression distribution of related genes, and different colors represent different groups. *p < 0.05, **p < 0.01, ***p < 0.001.

The expression of different iron-associated genes in cisplatin-sensitive cells (A2780) and cisplatin-resistant cells (A2780CP) was compared to investigate the relationship between DIMP and cisplatin resistance in ovarian cancer. A statistical difference was observed in the expression of 14 genes involved in iron import between A2780 and A2780CP (Fig. 4A). Specifically, among these 14 genes, the expression of 13 genes in iron import was lower in A2780CP than in A2780, indicating that inhibiting iron import might contribute to cisplatin resistance. The expression of *HIF1A*, *GPI*, and *ACO1* involved in iron regulation was higher while the expression of *MTF1* was lower, in A2780CP than in A2780 (Fig. 4B). These data indicate that activation of iron regulation may be associated with cisplatin resistance in ovarian cancer. The expression of most genes involved in iron utilization was higher in A2780CP than in A2780, except for *PPOX* and *ISCU* (Fig. 4C). These data indicate that activation of iron utilization may be associated with cisplatin resistance in ovarian cancer. The expression of the five genes involved in iron export was not significantly different between A2780CP and A2780 (Fig. 4D). The expression of *FTL* and *FTH1* involved in iron storage was lower in A2780CP than in A2780 (Fig. 4E). These data indicate that reduced iron storage and iron import may be associated with cisplatin resistance in ovarian cancer.

### Relationship between different iron metabolism processes and the prognosis of ovarian cancer

Our analysis revealed that most genes in iron import were associated with poor progression-free survival (PFS) of ovarian cancer, including *CYBRD1*, *SLC11A2*, *FECH*, *SLC25A28*, *LRP2*, *STEAP2*, *SLC22A17*, *HEPH*, *SLC39A14*, *SLC48A1*, *PCBP1*, and *PCBP2*, while the expression of *STEAP3* was associated with improved PFS (Table 2). The expression of *HFE*, *MTF1*, and *BMP6* involved in iron regulation was associated with improved PFS in ovarian cancer, whereas the expression of *HIF1A*, *GPI*, and *HAMP* involved in this process was associated with poor PFS in ovarian cancer (Table 2). The expression of most genes involved in iron export, including *ABCG2*, *FLVCR1*, *FLVCR2*, and *SLC40A1*, was associated with improved PFS in ovarian cancer (Table 2). The expression of *ALAS2* and *HMBS* involved in iron utilization was associated with improved PFS in ovarian cancer, whereas expression of *ABCB7*, *ALAD*, *FXN*, *UROS*, *ISCU*, and *CPOX* in this process was associated with poor PFS in ovarian cancer (Table 2). However, the expression of genes involved in iron storage was not significantly associated with the prognosis of ovarian cancer (Table 2). These data indicate that activation of iron import and iron utilization, and inhibition of iron export may be associated with poor PFS in ovarian cancer, whereas there are no relationships between iron regulation, iron storage, and PFS of ovarian cancer.

**Table 2:** Different iron metabolism processes (DIMP) and PFS of ovarian cancer

Iron metabolism process	Gene	HR	*p* value
Iron import	LCN2	0.92(0.81–1.04)	0.19
	CP	0.89(0.74–1.08)	0.23
	CYBRD1	1.54(1.27–1.86)	7.1e-06
	SLC11A2	1.19(1.05–1.36)	0.0086
	FECH	1.28(1.05–1.57)	0.013
	SLC46A1	0.84(0.68–1.04)	0.11
	SLC25A28	1.37(1.13–1.65)	0.001
	LRP2	1.48(1.19–1.84)	0.00036
	STEAP3	0.81(0.72–0.92)	0.0014
	STEAP2	1.49(1.19–1.86)	0.00041
	TFR2	1.07(0.94–1.21)	0.3
	TFRC	1.11(0.97–1.26)	0.13
	SLC22A17	1.25(1.09–1.43)	0.0014
	TF	1.1(0.97–1.26)	0.13
	HEPH	1.19(1.05–1.35)	0.0066
	SLC25A37	1.08(0.88–1.32)	0.46
	LTF	1.09(0.95–1.24)	0.21
	SLC39A14	1.27(1.12–1.44)	2e-04
	SLC48A1	1.18(1.03–1.35)	0.014
	PCBP1	1.17(1.02–1.34)	0.028
	PCBP2	1.27(1.1–1.47)	0.0013
Iron regulation	IREB2	1.24(1–1.52)	0.049
	HIF1A	1.16(1.01–1.34)	0.037
	HFE	0.86(0.75–0.97)	0.018
	GPI	1.39(1.22–1.58)	3.4e-07
	MTF1	0.7(0.58–0.84)	0.00016
	BMP6	0.78(0.69–0.89)	0.00022
	HAMP	1.21(1.05–1.39)	0.0099
	HMOX1	0.88(0.77–1)	0.048
	HMOX2	1.09(0.94–1.27)	0.26
	ACO1	0.87(0.76–1.01)	0.06
Iron export	ABCG2	0.87(0.77–0.99)	0.032
	FLVCR1	0.77(0.64–0.94)	0.0092
	FLVCR2	0.79(0.69–0.91)	0.0012
	SLC40A1	0.66(0.53–0.82)	0.00016
	MON1A	1.15(0.95–1.4)	0.15
Iron storage	FTL	0.87(0.76–1)	0.057
	FTH1	1.1(0.96–1.27)	0.17
Iron utilization	ABCB6	0.89(0.78–1.01)	0.069
	ABCB7	1.37(1.18–1.57)	1.6e-05
	ALAD	1.25(1.1–1.42)	0.00054
	ALAS1	1.09(0.96–1.24)	0.17
	ALAS2	0.8(0.7–0.91)	0.00093
	FXN	1.25(1.03–1.52)	0.025
	HMBS	0.86(0.75–0.99)	0.039
	PPOX	0.88(0.77–1.01)	0.06
	UROD	1.08(0.94–1.24)	0.27
	UROS	1.16(1.01–1.32)	0.031
	ISCU	1.17(1.02–1.34)	0.025
	ISCA1	1.15(1–1.32)	0.056
	CIAO1	1.09(0.95–1.25)	0.22
	CPOX	1.27(1.11–1.45)	0.00032

We also discussed the relationship between DIMP and OS of ovarian cancer. The expression of *CYBRD1*, *LRP2*, *TFRC*, *SLC22A17*, *HEPH*, *SLC39A14*, and *PCBP2* involved in iron import was associated with poor OS of ovarian cancer, whereas the expression of *LCN2*, *CP*, *SLC46A1*, *STEAP3*, and *LTF* in this process was associated with improved OS in ovarian cancer (Table 3). The expression of *MTF1* and *BMP6* involved in iron regulation was associated with improved OS in ovarian cancer, whereas the expression of *IREB2*, *GPI*, and *HMOX1* in this process was associated with poor OS in ovarian cancer (Table 3). The expression of *FLVCR2* and *SLC40A1* involved in iron export was associated with improved OS in ovarian cancer, while the expression of *MON1A* in this process was associated with poor OS in ovarian cancer (Table 3). The expression of *FLT* involved in iron storage was associated with poor OS in ovarian cancer (Table 3). The expression of *ALAS2*, *HMBS*, and *FXN* involved in iron utilization was associated with improved OS in ovarian cancer, whereas the expression of *ALAD*, *ALAS1*, and *CPOX* in this process was associated with poor OS in ovarian cancer (Table 3). These data indicate that increased iron storage is associated with poor OS in ovarian cancer, whereas there are no relationships between iron import, iron regulation, iron export, iron utilization, and OS in ovarian cancer.

**Table 3:** DIMP and OS of ovarian cancer

Iron metabolism process	Gene	HR	*p* value
Iron import	LCN2	0.82(0.71–0.94)	0.006
	CP	0.79(0.63–0.99)	0.043
	CYBRD1	1.38(1.12–1.69)	0.0026
	SLC11A2	1.1(0.95–1.26)	0.2
	FECH	1.22(0.95–1.56)	0.11
	SLC46A1	0.65(0.51–0.83)	0.00051
	SLC25A28	0.84(0.67–1.04)	0.11
	LRP2	1.37(1.12–1.69)	0.0027
	STEAP3	0.8(0.7–0.9)	0.00047
	STEAP2	1.15(0.94–1.41)	0.19
	TFR2	0.92(0.79–1.06)	0.24
	TFRC	1.32(1.15–1.51)	5.4e-05
	SLC22A17	1.23(1.08–1.39)	0.002
	TF	1.1(0.97–1.27)	0.13
	HEPH	1.27(1.11–1.46)	4e-04
	SLC25A37	0.86(0.7–1.07)	0.18
	LTF	0.84(0.72–0.97)	0.019
	SLC39A14	1.25(1.1–1.42)	0.00066
	SLC48A1	0.9(0.78–1.05)	0.18
	PCBP1	1.13(0.98–1.3)	0.096
	PCBP2	1.18(1.03–1.37)	0.021
Iron regulation	IREB2	1.25(1.02–1.54)	0.031
	HIF1A	1.14(0.99–1.32)	0.066
	HFE	0.89(0.77–1.02)	0.1
	GPI	1.38(1.21–1.57)	1.1e-06
	MTF1	0.73(0.59–0.9)	0.0034
	BMP6	0.84(0.74–0.95)	0.0074
	HAMP	0.89(0.78–1.01)	0.072
	HMOX1	1.21(1.05–1.39)	0.0096
	HMOX2	0.94(0.81–1.09)	0.41
	ACO1	0.94(0.82–1.07)	0.33
Iron export	ABCG2	0.91(0.8–1.04)	0.15
	FLVCR1	1.21(0.99–1.48)	0.069
	FLVCR2	0.82(0.71–0.94)	0.0048
	SLC40A1	0.69(0.55–0.87)	0.0014
	MON1A	1.29(1.05–1.6)	0.016
Iron storage	FTL	1.19(1.05–1.35)	0.0083
	FTH1	1.07(0.92–1.24)	0.4
Iron utilization	ABCB6	0.91(0.8–1.03)	0.14
	ABCB7	1.13(0.98–1.31)	0.095
	ALAD	1.18(1.04–1.35)	0.011
	ALAS1	1.21(1.06–1.38)	0.0035
	ALAS2	0.82(0.72–0.93)	0.0025
	FXN	0.81(0.66–0.99)	0.039
	HMBS	0.78(0.67–0.91)	
	PPOX	0.91(0.8–1.03)	0.14
	UROD	1.14(1–1.3)	0.046
	UROS	1.09(0.95–1.26)	0.22
	ISCU	1.1(0.95–1.28)	0.18
	ISCA1	1.13(0.99–1.28)	0.069
	CIAO1	1.1(0.95–1.27)	0.19
	CPOX	1.18(1.04–1.35)	0.01

### Prognostic predictive value of iron metabolism-associated genes in ovarian cancer

**Figure 5:**
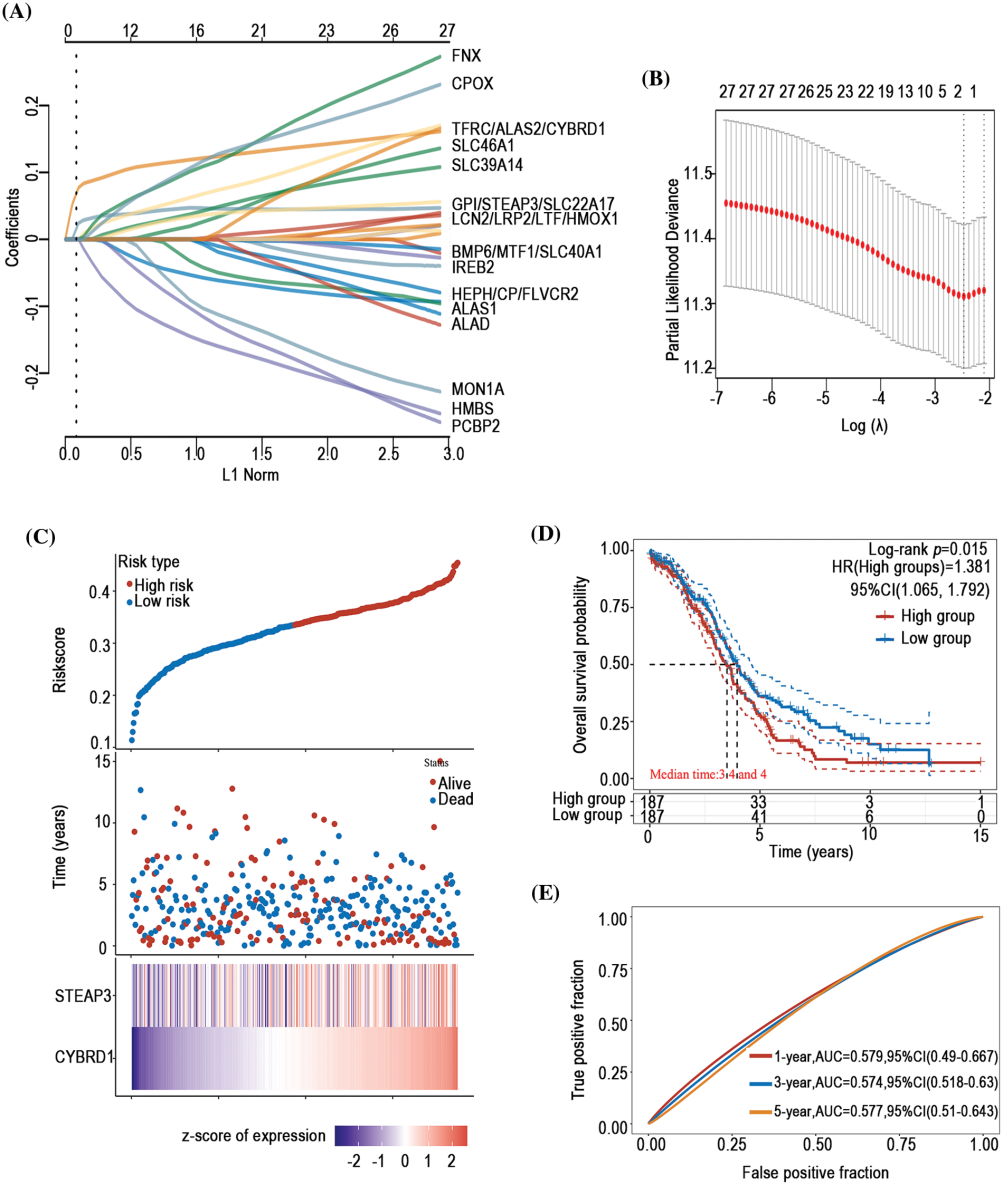
Establishment of an OS prognosis model using iron metabolism-associated genes. Using OS risk factors, an OS prognosis model was established with various iron metabolism-associated genes. (A) Coefficients of selected features are demonstrated by the lambda parameter. (B) Partial likelihood deviance *vs*. log (λ)was drawn using the LASSO Cox regression model. (C) Prognostic analysis of gene signature was conducted using a TCGA dataset in which the top represents the scatter graph of the Riskscore from low to high, and various colors represent various expression groups; The middle represents the scatter plot distribution of Riskscore corresponding to the survival time and survival state of various samples; The bottom graph represents a heat map of the genes in this signature. (D) Survival status of the patients was examined between the various groups, and more dead patients corresponded to a higher risk score. (E) Time-dependent ROC analysis of the gene signature was conducted.

**Figure 6:**
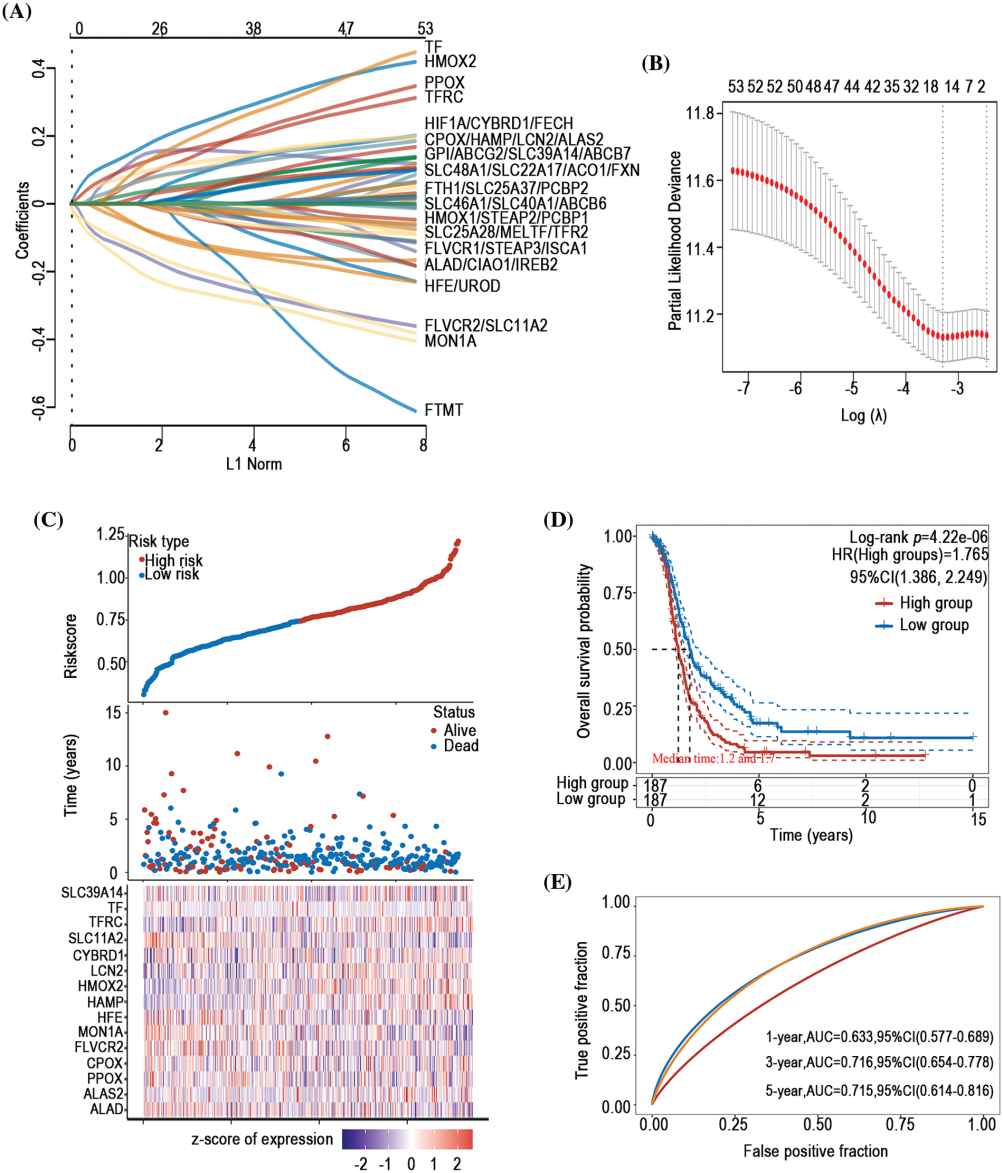
Establishment of a PFS prognosis model using iron metabolism-associated genes. With PFS risk factors, the PFS prognosis model was established with different iron metabolism-associated genes. (A) Coefficients of chosen features are demonstrated by the lambda parameter. (B) Partial likelihood deviance *vs*. log (λ)was drawn using the LASSO Cox regression model. (C) Prognostic analysis of gene signature was conducted using a TCGA dataset in which the top represents the scatter graph of the Riskscore from low to high, and different colors represent various expression groups; The middle represents the scatter plot distribution of Riskscore corresponding to the survival time and survival state of various samples; The bottom graph represents a heat map of the genes in this signature. (D) Survival status of the patients was examined between the different groups, and more dead patients corresponded to a higher risk score. (E) Time-dependent ROC analysis of the gene signature was conducted.

To investigate the prognostic predictive value of iron metabolism-associated genes in ovarian cancer, a prognosis model was developed with 376 ovarian cancer patients from The Cancer Genome Atlas (TCGA) database. For total survival, two genes were used to construct the OS prognosis model: Riskscore = (0.0539)*CYBRD1 + (0.002)*STEAP3 (lambda.min = 0.0844) (Figs. 5A and 5B). The OS of the high-risk group was shorter than that of the low-risk group (Figs. 5C and 5D). Furthermore, it was discovered that there was a certain prognosis predictive value in ovarian cancer with this risk model (Fig. 5E). A total of 15 genes were used to establish the PFS prognosis model: Riskscore = (−0.0459)*ALAD + (0.1011)*ALAS2 + (0.043)*PPOX + (0.0463)*CPOX + (−0.1241)*FLVCR2 + (−0.1622)*MON1A + (−0.0366)*HFE + (0.076)*HAMP + (0.1343)*HMOX2 + (0.0226)*LCN2 + (0.0485)*CYBRD1 + (−0.1091)*SLC11A2 + (0.0968)*TFRC + (0.0616)*TF + (0.0052)*SLC39A14 (Figs. 6A and 6B). Moreover, the PFS of the high-risk group was shorter than that of the low-risk group (Figs. 6C and 6D). This risk model has a better predictive capacity, particularly for 3-year and 5-year prognoses (Fig. 6E).

### TGF-β regulated different iron metabolism processes

**Figure 7:**
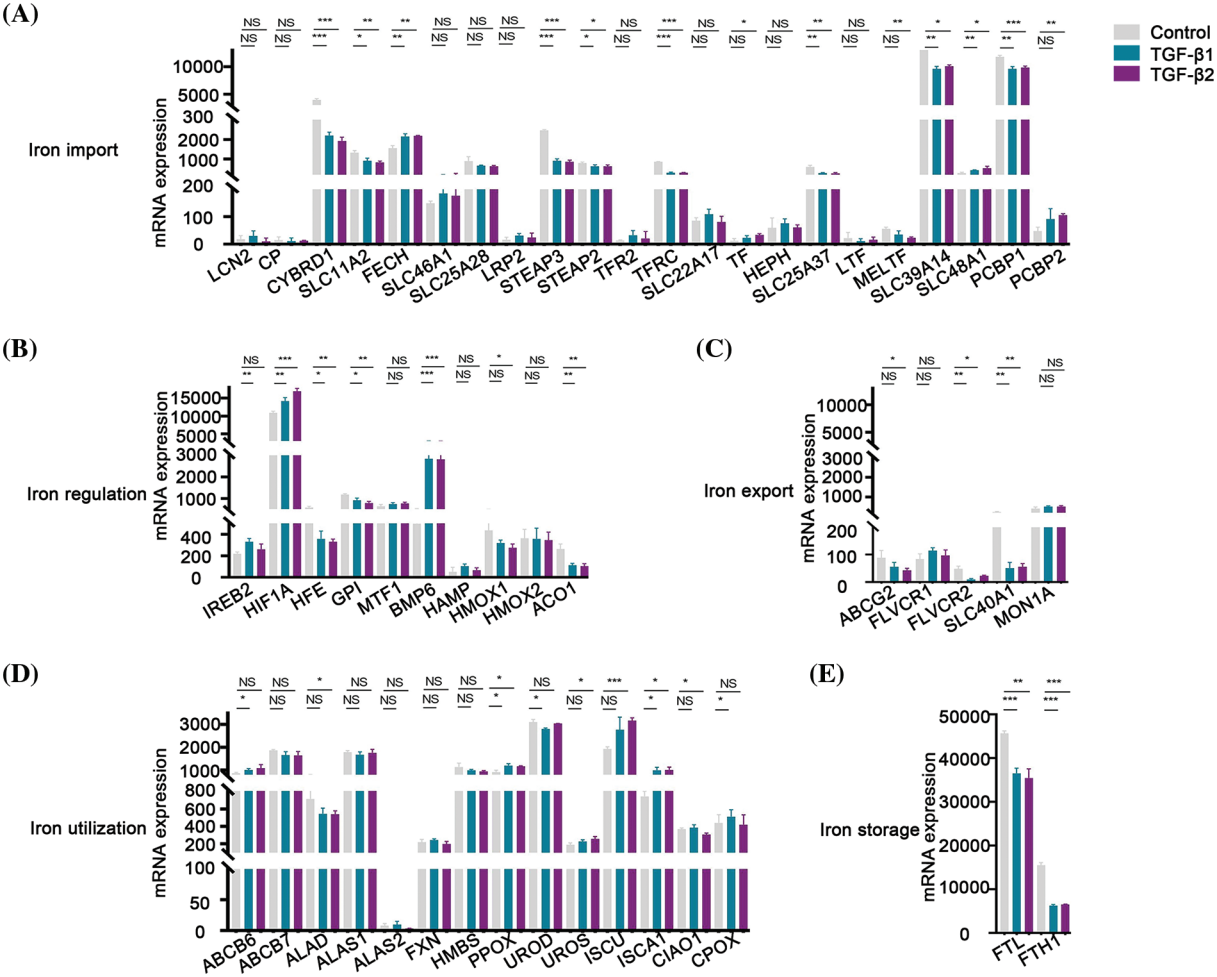
TGF-β inhibited the expression of different iron metabolism-associated genes. Treatment with 5 ng/ml TGF-β1 or TGF-β2 for 48 h, expression of DIMP-associated genes was compared between the TGF-β1 or TGF-β2 group and its control group, including iron import (A), iron regulation (B), iron export (C), iron utilization (D), and iron storage (E). The horizontal axis represents various genes, the vertical axis represents the expression distribution of related genes, and different colors represent various groups. *p < 0.05, **p < 0.01, ***p < 0.001.

**Figure 8:**
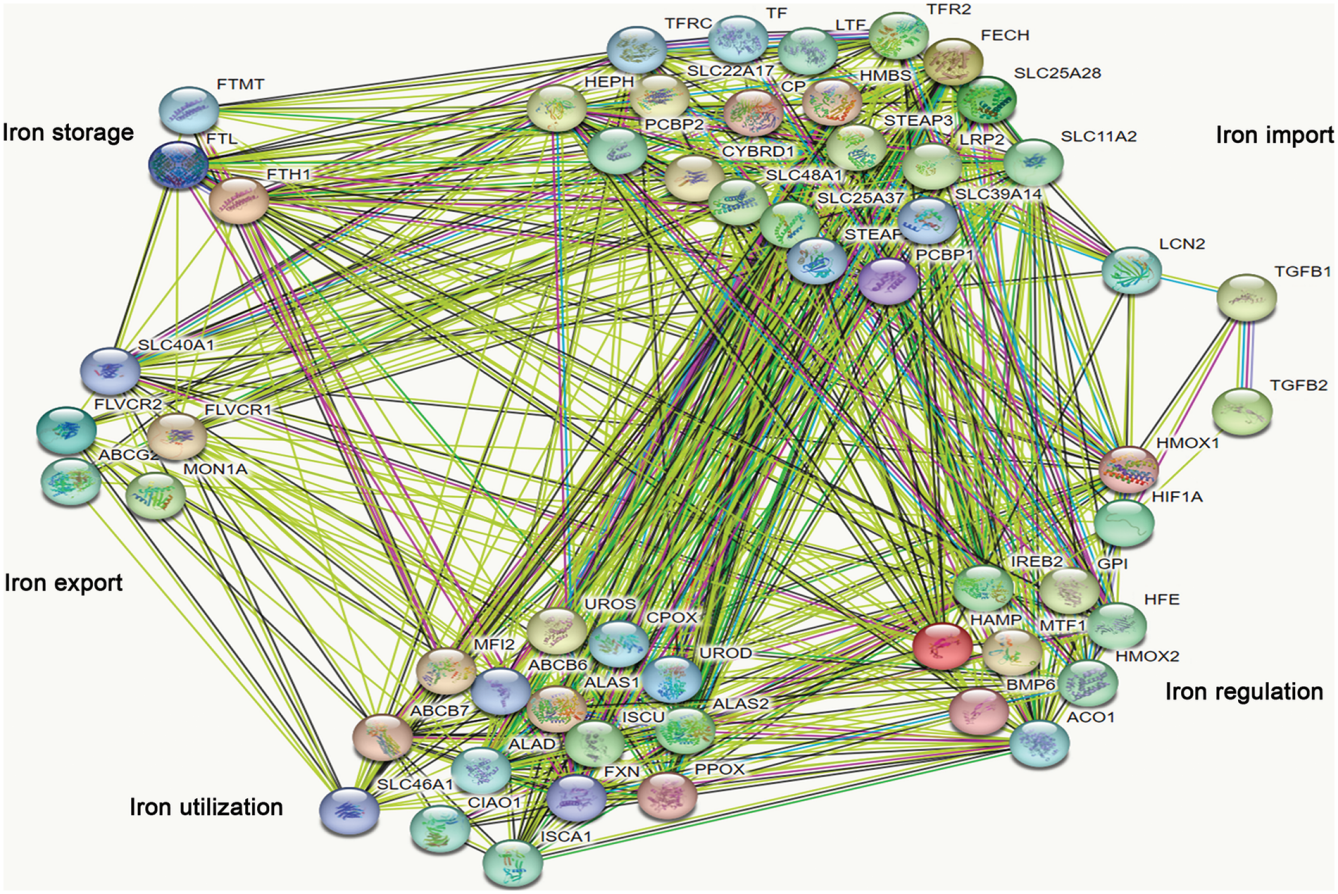
Analysis of the relationship between TGF-β1 or TGF-β2 and different iron metabolism-associated genes in ovarian cancer. With the STRING database, the relationship between TGF-β1 or TGF-β2 and various iron metabolism-associated genes was examined in ovarian cancer.

TGF-β was involved in the regulation of cellular processes such as differentiation, proliferation, and apoptosis. In this study, the expression of 8 genes involved in iron import was lower than that in the control group after being treated with TGF-β1 or TGF-β2 for 48 h, including *CYBRD1, SLC11A2, STEAP3, STEAP2, TFRC, SLC25A37, SLC39A14* and *PCBP1* (Fig. 7A). However, the expression of *FECH* and *SLC48A1* was higher after treatment with TGF-β1 or TGF-β2 than in the control group (Fig. 7A). These data show that inhibition of iron import by TGF-β1 or TGF-β2 may be responsible for the initiation, progression, drug resistance, and prognosis of ovarian cancer. The expression of *HFE, GPI*, and *ACO1* involved in iron regulation was lower than that of the control group after being treated with TGF-β1 or TGF-β2 (Fig. 7B). However, the expression of *HIF1A* and *BMP6* was higher than that in the control group (Fig. 7B). The relationship between TGF-β and iron regulation remains unclear based on the available data. The expression of *FLVCR2* and *SLC40A1* involved in iron export was lower than the control group after being treated with TGF-β1 or TGF-β2 (Fig. 7C). These data indicate that inhibition of iron export by TGF-β1 or TGF-β2 may be responsible for the initiation, progression, drug resistance, and prognosis of ovarian cancer. The expression of *PPOX* and I*SCA1* involved in iron utilization was higher than the control group after being treated with TGF-β1 or TGF-β2 (Fig. 7D). The relationship between iron utilization and TGF-β is still uncertain due to limited evidence. The expression of *FTL* and *FTH1* involved in iron storage was lower in TGF-β1 or TGF-β2 group than in the control group after being treated with TGF-β1 or TGF-β2 (Fig. 7E). These data show that inhibition of iron storage by TGF-β1 or TGF-β2 may be responsible for drug resistance of ovarian cancer. We also determined the mechanism by which TGF-β regulated iron metabolism-associated genes using the STRING database (https://cn.string-db.org/). Interestingly, we found interactions between TGF-β1 and LCN2, HMOX1, and HIF1A (Fig. 8). We also showed interactions between TGF-β2 and HIF1A, TGF-β1 (Fig. 8). Through LCN2, HMOX1, and HIF1A, TGF-β1 or TGF-β2 indirectly influence the function and activity of other proteins in DIMP, including those involved in iron import, iron regulation, iron storage, iron utilization, and iron export (Fig. 8).

## Discussion

In this study, we observed a compelling association between the inhibition of iron regulation, iron utilization, and iron export and the onset of ovarian tumors. Additionally, a surge in iron storage corresponded with the onset of these tumors as well. These observations suggest the diverse roles for DIMP in the initiation of ovarian cancer, which have not been reported previously, to the best of our knowledge. Moreover, the inhibition of iron utilization, inhibition of iron export, and increased iron storage can lead to both anemia and cellular iron overload, indicating that anemia and intracellular iron overload may be risk factors for ovarian cancer. Notably, anemia, characterized by low hemoglobin levels, has been recognized as an independent prognostic factor for ovarian cancer. These have been further supported by previous studies showing that iron overload is a crucial reason for ovarian cancer [70]. The precise nature of the link between anemia and the onset of ovarian cancer, however, remains an area of uncertainty. Our findings provide a promising avenue for further investigation of it in ovarian cancer.

Cisplatin resistance in ovarian cancer is a complex process, with numerous genes influencing the process. Recent studies have demonstrated that abnormal iron metabolism is related to cisplatin resistance [8,9]. Furthermore, Salatino et al. highlighted H-Ferritin as a key protein associated with cisplatin resistance in ovarian cancer [71]. Remarkably, treatment with an iron chelator has been shown to enhance the sensitivity of ovarian cancer cells to cisplatin [9]. Furthermore, a normal baseline hemoglobin level is deemed a substantial predictor of chemotherapy efficacy [72]. In this study, we showed that inhibition of iron import and iron storage contribute to cisplatin resistance. Similarly, the activation of iron regulation and iron utilization also leads to cisplatin resistance. These observations underscore the diverse roles of DIMP in modulating cisplatin resistance. Notably, inhibition of iron import, inhibition of iron storage, or activation of iron utilization can lead to anemia. This aligns with our predisposition that anemia could be a potential contributor to cisplatin resistance, which is consistent with previous studies [72]. Thus, ameliorating anemia conditions and restoring iron homeostasis in ovarian cancer may be a new approach to reverse cisplatin resistance.

The prognosis of ovarian cancer is influenced by multiple factors, including tumor type, stage, and many other variables. Anemia before chemotherapy in ovarian cancer patients has been considered a predictor of poor prognosis [73]. Altman AD identified hemoglobin levels higher than 80 g/l during chemotherapy signify a more favorable prognosis in ovarian cancer [74]. However, as a crucial component for hemoglobin synthesis, the relationship between DIMP and the prognosis of ovarian cancer has not been explored until now. In this study, we intriguingly discern that DIMP plays diverse roles in the prognosis of ovarian cancer. Specifically, our findings suggest that patients exhibiting activated iron import and utilization, or inhibited iron export tend to have poor PFS. Moreover, increased iron storage in patients correlates with poor OS, consistent with previous studies, indicating cellular iron overload as a negative prognostic indicator in ovarian cancer [8,9]. Importantly, we constructed an ovarian cancer prognosis model, which shows a superior prognostic predictive ability compared to previous prognosis models [75]. However, our model has a relatively low area under curve (AUC), which underscores the need for refining this model using extensive clinical data in the future.

Gene mutations or abnormal gene expression have recently been linked as potential causal factors for ovarian cancer. In our study, we observed a prevalent mutation in genes associated with iron metabolism. Notably, these mutations correlated positively with the pathological grade, lymphovascular invasion, and primary treatment response in ovarian cancer. This suggests mutations in iron metabolism-associated genes might serve as prognostic factors of ovarian cancer, which is a novel observation that has not been published previously.

TGF-β plays a dual role in cancer. While it has been shown to restrain cell proliferation and increase cell apoptosis in early-stage ovarian cancer [76], TGF-β also amplifies cell invasion and metastasis in advanced ovarian cancer [77]. Despite the potential therapeutic benefits of targeting TGF-β for ovarian cancer treatment [78], its mechanistic roles, especially concerning chemo-resistance, remain elusive. Our findings suggest that inhibition of iron import, iron export, and iron storage may modulate TGF-β’s role in the initiation of ovarian cancer. Our protein interaction analysis further demonstrated potential connections between TGF-β and LCN2, HMOX1, or HIF1A, thus linking TGF-β signaling pathway to DIMP. This suggests that TGF-β may regulate DIMP through LCN2, HMOX1, or HIF1A in ovarian cancer. These insights present a promising avenue for combined therapies targeting both TGF-β and iron metabolism for ovarian cancer treatment. For instance, while TGF-β targeted agents like Trabedersen and Galunisertib have shown promise in combating ovarian cancer [79], DFO, as an iron chelator, is considered to be a tumor suppressor for the disease as well [8]. Combining DFO with Trabedersen or Galunisertib might amplify their anti-tumor effects, though this requires validation in further clinical trials.

DIMP have diverse roles in the onset and chemo-resistance of ovarian cancer, significantly impacting its prognosis. Understanding the role of DIMP in ovarian cancer can guide more precise interventions, potentially curbing the initiation and improving the prognosis of ovarian cancer. This study also unveils novel prognostic predictive factors for ovarian cancer, broadening the options for its management. Notably, our study indicates TGF-β as a promising therapeutic target, giving its regulation on the expression of multiple iron metabolism-associated genes. This opens the avenue for a combined therapeutic approach using TGF-β inhibitors and iron chelators in treating ovarian cancer.

## Data Availability

All the study data are included in the article.
